# P092: Antibiotic usage and appropriateness for a university hospital in Turkey: point prevalence results

**DOI:** 10.1186/2047-2994-2-S1-P92

**Published:** 2013-06-20

**Authors:** H Gül, A Karakaş, C Artuk, G Özbek, S Kılıç, CP Eyigün

**Affiliations:** 1 Infectious Diseases and Clinical Microbiology, Ankara, Turkey; 2 Infection Control, Gülhane Military Hospital, Ankara, Turkey

## Objectives

This study aims to determine antibiotic usage rates, causes of antibiotic usage and inappropriate usage rates in a university hospital with a 1,200-bed capacity.

## Methods

The study assessed antimicrobial drug usage among all hospitalised patients in the hospital on April 20, 2012, using the point prevalence method. Data were recorded using pre-prepared forms. Appropriateness of antibiotic usage was determined according to the appropriateness for the cause of antibiotic usage, the spectrum of the chosen antibiotics, usage dose, dose frequency and time of usage.

## Results

Of 666 patients staying in the hospital on the day of study, 262 (39.7%) were on antibiotics. Of those, 145 (55.3%) were on surgical wards, 98 (37.4%) were on medical wards and 19 (7.3%) were on paediatric wards. Of those 262 patients, 157 (59.9%) were taking only one type of antibiotic, 79 (30.2%) were taking two and 26 (9.9%) were taking three or more types of antibiotic. Antibiotic usage was appropriate in 55.7% and inappropriate in 44.3%. The inappropriate antibiotic usage rate was 75.9% among patients on surgical wards. The most common cause of inappropriate usage was unnecessarily long prophylaxis time (68.2%). Inappropriate antibiotic usage was found in 24 (24.5%) patients out of 98 patients on medical wards. When the causes of antibiotic usage were analysed, it was found that the cause of antibiotic usage was infection in 36.2%, prophylactic in 35.9%, and empirical in 27.9%. On the day the study was conducted, 367 antimicrobial drug were prescribed to 262 patients. The drugs most commonly prescribed were antibiotics from the cephalosporin (27.0%) and fluoroquinolone (20.2%) groups. When the diagnosis of 95 patients who were on antibiotics due to infection was reviewed, the most common infections were respiratory tract infections (37.9%), urinary system infections (12.6%), upper respiratory tract infections (8.4%), bloodstream infections (6.3%) and prosthesis infections (6.3%).

## Conclusion

Inappropriate antibiotic usage rates in surgical wards were high. This inappropriate usage is especially related to prophylaxis time. This is why it is necessary for surgeons to be educated regarding prophylactic antibiotic usage and to stick to the surgical prophylaxis guidelines.

## Disclosure of interest

None declared

